# The relationship between sleep deprivation and the worsening of mood disorders in health professionals working night shifts

**DOI:** 10.1590/1980-5764-DN-2024-0186

**Published:** 2025-05-19

**Authors:** Alexandre Almeida da Silva, Bianca Araujo Vieira, Júlio César Claudino dos Santos

**Affiliations:** 1 Centro Universitário Christus, Faculdade de Fisioterapia, Fortaleza CE, Brazil.; 2 Universidade Federal do Ceará, Fortaleza CE, Brazil.; 3 Centro Universitário Christus, Faculdade de Medicina, Fortaleza CE, Brazil.; 4 Centro Universitário Unifacvest, Lages SC, Brazil.

**Keywords:** Sleep Wake Disorders, Stress, Psychological, Sleep Deprivation, Mood Disorders, Transtornos do Sono-Vigília, Estresse Psicológico, Privação do Sono, Transtornos do Humor

## Abstract

Although the impact of sleep deprivation on the development of psychological disorders is widely recognized, little is known about the longitudinal relationship between sleep quality and mood disorders, specifically among health professionals.

**Objective:**

The objective of this study was to investigate the relationship between sleep quality and the incidence of mood disorders among health professionals who work night shifts, focusing on anxiety, depression, and stress.

**Methods:**

This is an observational, cross-sectional, and quantitative study, approved by the Ethics Committee of the Christus University Center (Unichristus), in accordance with Resolution 466/12. It was carried out using an online questionnaire via Google Forms, in the city of Fortaleza, Ceará.

**Results:**

A total of 45 health professionals from different areas took part in the survey, including nursing technicians, physiotherapists, nurses, and doctors. The results indicated a high incidence of sleep disorders, especially among nursing staff, with average scores of 11–13 on the Pittsburgh Sleep Quality Index (PSQI). The correlations found between sleep quality (as measured by the PSQI) and the symptoms of depression, anxiety, and stress were weak to moderate, with correlation coefficients (r) of 0.37, 0.44, and 0.48, respectively.

**Conclusion:**

The results suggest that there is an urgent need for interventions to improve sleep quality and reduce stress among health professionals working night shifts. Despite the promising findings, the study recommends further research to explore these relationships in more depth.

## RESUMO.

Embora o impacto da privação de sono sobre o desenvolvimento de transtornos psicológicos seja amplamente reconhecido, pouco se sabe sobre as relações longitudinais entre a qualidade do sono e os transtornos de humor especificamente entre profissionais da saúde.

**Objetivo:**

Investigar a relação entre a qualidade do sono e a incidência de transtornos de humor entre profissionais da saúde que trabalham em plantões noturnos, focando em ansiedade, depressão e estresse.

**Métodos:**

Este é um estudo observacional, transversal e quantitativo aprovado pelo Comitê de Ética do Centro Universitário Christus (Unichristus), conforme a Resolução nº 466/12. Foi realizado por meio de um questionário *online* via Google Forms, na cidade de Fortaleza, Ceará.

**Resultados:**

Participaram da pesquisa 45 profissionais de saúde de diversas áreas, incluindo técnicos em Enfermagem, fisioterapeutas, enfermeiros e médicos. Os resultados indicaram alta incidência de distúrbios do sono, especialmente entre a equipe de enfermagem, com pontuações médias de 11–13 no Índice de Qualidade do Sono de Pittsburgh (PSQI). As correlações encontradas entre a qualidade do sono (medida pelo PSQI) e os sintomas de depressão, ansiedade e estresse foram de fracas a moderadas, com coeficientes de correlação (r) de 0,37, 0,44 e 0,48, respectivamente.

**Conclusão:**

Os resultados sugerem que há a necessidade urgente de intervenções para melhorar a qualidade do sono e reduzir o estresse entre os profissionais da saúde que trabalham em plantões noturnos. Apesar das descobertas promissoras, o estudo recomenda a realização de mais pesquisas para explorar essas relações de maneira mais aprofundada.

## INTRODUCTION

Sleep is a natural process that is essential for the restoration and functioning of the brain, and is considered a vital and dynamic mechanism present in most species^1^. It plays an essential role in the emotional, physical, and cognitive development of individuals^2^, encompassing several fundamental functions for the human organism, such as regulating brain activities related to emotions and facilitating the processing and consolidation of memories^3^. In addition, proper sleep regulation is crucial for the balance of physiological systems, such as the cardiovascular^4^, immune^5^, and endocrine^6^ systems, with a direct influence on the nervous system^7^.

In addition, the ideal duration of sleep varies between 7 and 9 h^8^, alternating between two distinct neurophysiological states: rapid eye movement (REM) sleep and non-rapid eye movement (NREM) sleep^9^. REM sleep is characterized by REMs and cortical activation similar to the waking state in sleeping individuals^10^ and is also involved in emotional processing^11^. NREM sleep, especially during the slow-wave phase (SWS), shows a reduction in high-frequency electrical activity in multiple neural networks. This process is related to memory consolidation^12^ and physical recovery^13^.

Sleep deprivation can be described as changes in the pressure to sleep, related to the homeostatic process, and in the pressure to stay awake, associated with the circadian cycle^14^. Disturbances in REM sleep caused by deprivation can activate the sympathetic nervous system and the hypothalamic-pituitary-adrenal axis, leading to immunological changes in the central nervous system (CNS). This increases the production of pro-inflammatory factors, such as tumor necrosis factor (TNF)-α and interleukin (IL)-6^15^. Other impacts of chronic sleep deprivation include a decline in physical, mental, emotional, and behavioral functions^16^, as well as daytime sleepiness, which reduces the speed of response to unexpected changes, increasing the risk of errors and accidents^17^.

Sleep deprivation causes a decline in cognitive functions during wakefulness, impairing decision-making abilities^18^. Thus, night-shift professionals are more susceptible to stress and fatigue^19^, predisposing them to depression and anxiety, which affect workplace interactions and individual behavior^20^. Higher levels of burnout, depression, and suicidal ideation have been reported among sleep-deprived night-shift health professionals^21,22^.

Recent studies^23-25^ show the prevalence of mood disorders, including anxiety, depression, and burnout, among healthcare workers subjected to sleep deprivation due to increased nighttime hours during the COVID-19 pandemic. Research indicates that sleep deprivation in health professionals is associated with deficits in decision-making capacity, including risk–benefit analysis in clinical procedures^26^, leading to greater susceptibility to errors with serious implications for patient safety^18^.

Sleep deprivation among health professionals negatively affects their neurobehavioral performance, compromising the quality of patient care. Long shifts are associated with a higher risk of substance use, unethical behavior, and psychological distress, especially due to frequent night shifts and irregular changes in working hours^27^. Lack of sleep impairs memory, reduces cognitive ability, and interferes with decision-making, leading to an increase in errors among professionals. These failures compromise safe practices and the quality of care in clinical environments^28^.

The use of scales and questionnaires is essential for assessing sleep quality. These include the Pittsburgh Sleep Quality Index (PSQI), used to measure the quality and quantity of sleep in the last month^29^. Another important tool is the Depression, Anxiety and Stress Scale (DASS), validated for Portuguese and adapted to the Brazilian sociocultural context. With 21 items and answers ranging from 0 to 3, the DASS assesses clinical conditions such as depression, anxiety, and stress, which shares characteristics such as negative effects, emotional distress, and neurophysiological changes in the hypothalamic-pituitary-adrenal axis^30,31^.

Therefore, the implications of sleep deprivation are evidenced in the scientific literature as affecting numerous brain, subcortical, and cortical regions. Changes in the connectivity of brain areas as a result of sleep deprivation influence the development of disorders such as anxiety, depression, and even burnout syndrome, which are prevalent in today's society. This study aimed to analyze the relationship between sleep quality and the prevalence of mood disorders in health professionals who work night shifts.

## METHODS

### Type of research

This research, approved by the Ethics Committee of the Christus University Center (Unichristus) under Resolution 466/12 (CAAE: 67721023.5.0000.5049), is an observational, cross-sectional, and quantitative epidemiological study. Conducted in Fortaleza, Ceará, it used an online questionnaire via Google Forms. The sample comprised 45 night-shift healthcare professionals. Participants of both genders, aged 18 years or older, and working at least one night shift per month were included. All participants provided informed consent prior to inclusion in the study.

Two validated instruments were used: the PSQI and the DASS-21. The questionnaire links were shared through WhatsApp groups to reach the target population.

### Statistical analysis

Data were tabulated using Microsoft Excel (version 13) and analyzed with JAMOVI (version 2.3.13). The normality of continuous variables was assessed using the Shapiro-Wilk test. Descriptive statistics summarized prevalence and sociodemographic data, employing measures such as mean and standard deviation for normal distributions.

The Shapiro-Wilk test was used to verify the normality of the distributions of the numerical variables. This test is widely used due to its sensitivity, especially in small-to-moderate samples, to assess whether the data follow a normal distribution, which is a necessary assumption for the application of parametric tests, such as Pearson's correlation test. Pearson's correlation test is appropriate for identifying possible linear associations between variables, such as sleep quality (measured by the PSQI) and mood scores (measured by the DASS-21), as long as the normality and linearity conditions are met. For data that did not meet the normality assumptions, the Spearman correlation test was applied, a non-parametric alternative to the Pearson test.

Spearman's correlation test was used in this study and was selected due to its suitability when the variables of interest did not have a normal distribution, i.e., a p-value of <0.05, and when the data were ordinal in nature. This method assesses the monotonic relationship between two variables, thus making it possible to identify patterns of association that do not necessarily follow a linear relationship. The Spearman correlation test was chosen to guarantee the validity and reliability of the conclusions presented, thus contributing to the quality and relevance of the results obtained in the context of this study.

Pearson's correlation test is also used in the parametric analysis to measure two variables that are linearly related. In this way, the correlation assumes that the variables in question are normally distributed, i.e., the value p>0.05. Pearson's correlation test was carried out in this study between the number of shifts and the variables of sleep quality and stress levels among professionals. The strength of correlations was categorized as follows: very strong (≥0.9), strong (0.7–0.89), moderate (0.4–0.69), weak (0.2–0.39), and very weak (0.0–0.19)^32^.

### Dependent variables

The PSQI consists of 19 questions, of which 9 are used in research investigation and assess the 7 components of sleep in a quantitative way, while the other 10 are used to provide qualitative insights that can be useful in clinical analysis. The DASS-21 is made up of three subscales –– depression, anxiety, and stress –– each scored on a 4-point scale (0–3). The results are added together and multiplied by two, classifying severity as normal, mild, moderate, severe, or extremely severe^31^.

## RESULTS

Data were collected between July and December 2023. A total of 48 health professionals took part in the study; however, three of them did not report working night shifts during the collection period, resulting in their exclusion. Therefore, the total sample was 45 participants. The average age of the individuals involved was 33.5 years, with a range between 21 and 60 years (Table 1).

**Table 1:** Descriptive statistical analysis.

	Stress	Anxiety	Depression	PSQI	Number of shifts
N	45	45	45	45	45
W for Shapiro-Wilk	0.966	0.905	0.918	0.968	0.954
p for Shapiro-Wilk	0.211	0.001	0.004	0.245	0.071

Approximately 71% of the participants included in the study were female, a third of whom were nursing technicians. The number of night shifts observed ranged from 3 to 25, with an average of 12.7 shifts. It is noteworthy that health professionals classified as nursing technicians had the highest average number of night shifts, with a value of 14.5, surpassing even the general average.

The participants involved in this investigation predominantly carried out their activities on duty in public hospital institutions in the city of Fortaleza. In contrast, only eight participants reported working shifts in private hospitals, while four professionals worked in both public and private hospitals.

When the study variables were analyzed using the Shapiro-Wilk test, it was found that the number of shifts (p>0.07), the PSQI (p>0.24), and stress symptoms (p>0.21) showed values above p>0.05. Therefore, Pearson's correlation test was used to compare these variables. Then, using the same normality test (Shapiro-Wilk), the anxiety (p<0.001) and depression (p<0.004) variables showed values lower than 0.05, indicating that the variables did not exhibit a normal distribution, so Spearman's correlation test was used (Supplementary Material: https://www.demneuropsy.com.br/wp-content/uploads/2025/01/DN-2024.0186-Supplementary-Material.docx).

### Evaluation of the Pittsburgh Sleep Quality Index in night-shift workers

**Figure 1:**
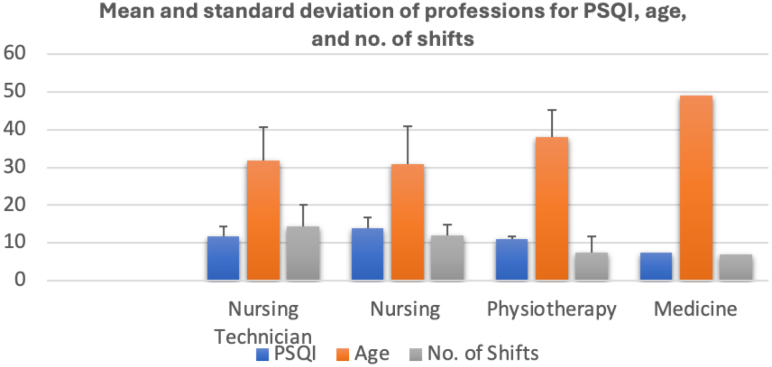
Sleep quality analysis.

The health professionals who took part in the study showed significant changes in sleep quality. Three of the four categories analyzed had an average of more than 10 on the PSQI, indicating the presence of sleep disorders among these professionals. The category of medical professionals showed an average of 7.5 on the index (Figure 1), resulting in poor sleep quality. Therefore, all the participating professionals had a range between sleep disorders and poor sleep quality.

The number of shifts, age, and nature of profession are some of the factors that can influence the quality of sleep of these professionals. It is therefore important to emphasize that nursing professionals and nursing technicians were the categories with the highest average number of night shifts per month 12. ≥12 Based on these observations, the changes in sleep patterns observed through the evaluation of the PSQI among individuals under these professional categories were interpreted as pathological manifestations. The correlation observed between the number of shifts and the variables of sleep quality (r=0.28) and stress level (r=0.12) among the professionals was assessed as being of weak magnitude (Table 2). These results indicate that the relationship between the number of shifts and sleep quality does not show a substantially significant association. However, it is important to point out that these conclusions should be interpreted with caution due to the small sample size, which limits the generalization of the findings to the entire study population.

**Table 2:** Correlation matrix using Pearson's correlation test between the number of shifts and the quality of sleep (PSQI), the number of shifts and the level of stress (DASS-21), and Correlation matrix between the number of shifts and the level of anxiety and depression (DASS-21) using Spearman's correlation test.

		PSQI	Number of shifts	Anxiety
Number of shifts	Pearson's R	0.280	-	0.197
	gl	43	-	43
	p-value	0.062	-	0.194
Stress	Pearson's R	-	0.125	-
	gl	-	43	-
	p-value	-	0.413	-
Depression	Spearman's Rho	-	0.050	0.699
	gl	-	43	43
	p-value	-	0.743	<0.001

### Evaluation of the Depression, Anxiety and Stress Scale-21 in night-shift workers

**Figure 2:**
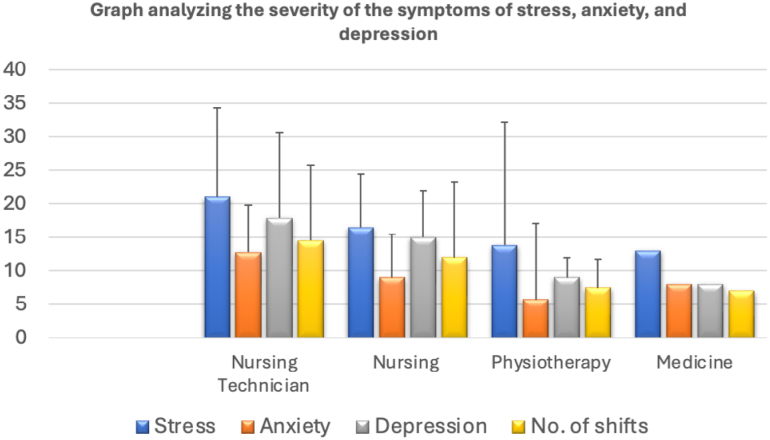
Analysis of the severity of the symptoms of stress, anxiety, and depression with respect to different medical professions.

With regard to the level of stress shown by the professionals analyzed, the nursing technicians showed an average indicating moderate stress, while others showed an average characterized by mild levels of stress. While analyzing the symptoms of anxiety and depression, the participants showed an average ranging from normal to moderate (Figure 2). However, the nursing technicians stood out as having higher levels of anxiety and depression symptoms compared to the other professional categories. To this end, the high incidence of the symptoms of stress, anxiety, and depression may be related to various factors, including the frequency of night shifts, the nature of the profession, and the quality of their sleep.

The correlation between the number of shifts and the levels of depression (r=0.05) and anxiety (r=0.19) was assessed as being of weak magnitude (Table 2). These results suggest that the relationship between the number of shifts and the levels of depression and anxiety does not show a substantially significant association. It is therefore crucial to stress that these conclusions should be interpreted with caution, given the small sample size, which restricts the generalization of the findings to the entire target population.

### Correlation between the Pittsburgh Sleep Quality Index and the Depression, Anxiety and Stress Scale-21

While analyzing the correlation between the PSQI and the DASS-21, a weak association was found between sleep quality and symptoms related to depression. However, sleep quality showed a moderate correlation with the symptoms of anxiety and stress. These findings suggest the possibility of a significant sample limitation, which may influence the generalizability of the results to all health professionals in Fortaleza. However, the moderate correlation identified provides encouraging evidence on this subject.

**Figure 3:**
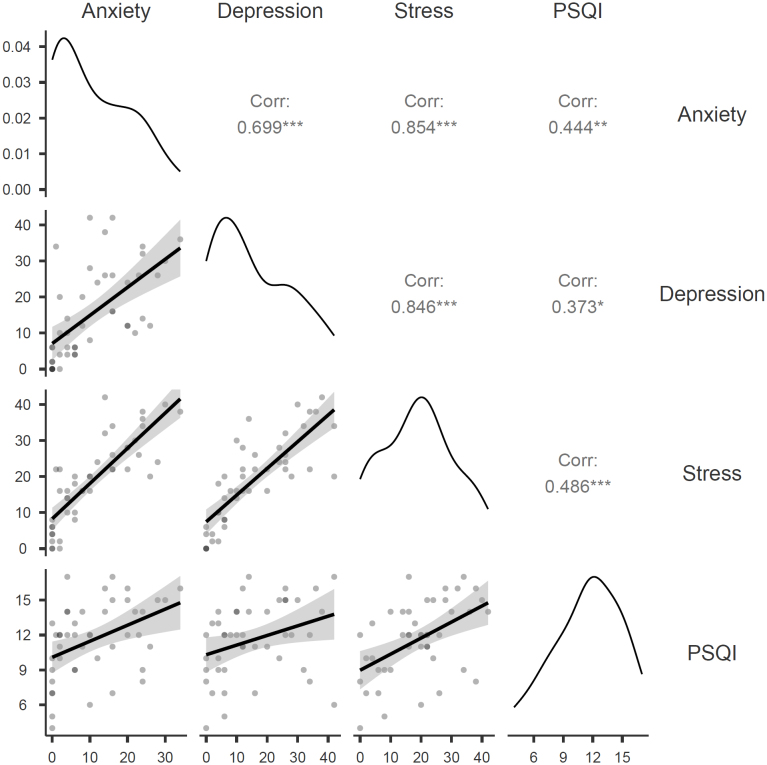
Graph of correlations between PSQI and DASS-21 scales.

The presence of substantial correlations between professionals’ stress levels and the symptoms of depression (r=0.846) and anxiety (r=0.854) is notable, pointing to a strong correlation (Figure 3). The findings suggest that the higher the stress levels, the more pronounced the symptoms of depression and anxiety among health professionals.

The correlation of sleep quality with the symptoms of depression, anxiety, and stress measured by the PSQI showed a weak-to-moderate correlation (Figure 3). Therefore, the worse the quality of sleep, the worse the professionals’ symptoms of depression, anxiety, and stress.

## DISCUSSION

In this study, we found that sleep deprivation in healthcare professionals has a direct impact on sleep quality, which, together with long working hours, is directly associated with an increased risk of developing the symptoms of depression, anxiety, and stress among night-shift workers. While previous studies have individually analyzed the relationship between sleep quality and depression in professional categories, this research is the first to analyze the increased risk of sleep deprivation for the development of depression, anxiety, and stress in a multi-professional way, in four professional categories. We also found that the chronicity of sleep deprivation, whether partial or total, among professionals can lead to significant symptoms of sleep disorders.

Health professionals who had sleep disorders, measured by the PSQI, had greater symptoms of stress, followed by anxiety and depression, quantified by the DASS-21, which demonstrates the directly proportional correlation between sleep disorders and increased depressive, anxious, and stressful symptoms. This finding is consistent with previous studies which showed that the presence of sleep disorders, such as insomnia, obstructive sleep apnea, and hypersomnia, was associated with physical and psychological stress^32,33^. In addition, it is important to note that the professional category of nursing technicians was the one that worked the most night shifts and, consequently, the one with the worst quality of sleep and the greatest severity of symptoms related to stress, anxiety, and depression.

Overall, our results are consistent with studies showing that sleep deprivation in health professionals has been associated with high levels of psychiatric symptoms, such as depression, anxiety, and stress, especially when concomitantly related to an extensive night workload^34,35^. Our data indicate that there is a directly proportional correlation, albeit weak, between sleep quality and mood disorders, i.e., the worse the sleep disturbances, the greater the depressive, anxious, and stressful symptoms in these professionals. In addition, there is no significant distinction between the etiological roles of sleep disorders and sleep deprivation in the context of the development of depression, anxiety, and stress, regardless of the presence or absence of a previous history of depression, i.e., it was observed that sleep deprivation can potentiate the progression of mood disorders.

Our findings are consistent with research showing that sleep deprivation has a degrading effect on the various dimensions of neurobehavioral function, ranging from the performance of psychomotor vigilance to aspects such as the affective and emotional state of individuals^36,37^. In this context, the findings are in line with studies conducted in different populations, which show an association between sleep deprivation and poor sleep quality with the manifestation of depression, anxiety, and stress^38,39^. It is important to note that Kalmbach et al.^35^ in a prospective cohort study identified that every hour less of sleep for medical residents corresponded to a 27% increase in the chances of making mistakes in the practice of their profession. In addition, residents without depression had a medical error rate of 15% and residents with chronic depression had an error rate of 41%. These results highlight the impact of sleep disorders and long night shifts on the mental health of healthcare professionals.

Sleep-deprived individuals show a fragmentation of REM sleep, a crucial phase for emotional memory processing^40^. Sleep deprivation is closely related to changes in the connectivity of brain areas, such as the amygdala, dorsolateral prefrontal cortex (DLPFC), thalamus, medial prefrontal cortex (mPFC), and posterior cingulate cortex (PCC). These alterations are associated with an increase in oxidative stress in neurons, which occurs due to the accumulation of reactive oxygen species (ROS) resulting from high cellular metabolism and a decrease in antioxidant defenses. This oxidative stress compromises the integrity of neuronal cell membranes, causing damage to critical structures such as mitochondria and affecting biochemical processes that are essential for sleep stability^41,42^. In this way, the integrity and continuity of the sleep phases, which are fundamental to emotional and cognitive balance, are compromised by sleep deprivation, generating a greater likelihood of unstable task performance and susceptibility to errors in healthcare professionals^43^.

In addition to errors from sleep and mood disorders, long-term sleep deprivation increases oxidative stress, threatening the health of night-shift professionals and their patients. Previous studies^44,45^ have shown that sleep deprivation raises neuroinflammatory biomarkers such as TNF, IL-6, and β-amyloid protein, which is linked to Alzheimer's disease. Our results suggest that promoting adequate sleep among night-shift healthcare professionals can help prevent depression, anxiety, stress, and neurodegenerative diseases, benefiting both patients and professionals. Thus, professionals with sleep disorders should be prioritized for preventive interventions and early treatment, such as cognitive-behavioral therapy (CBT), for insomnia.

This study has limitations that must be considered. The small sample size restricts the generalization of the results. Additionally, the electronic distribution of the questionnaire may introduce a response bias. Full diagnostic assessments for depression, anxiety, and stress require clinical interviews by specialized professionals, beyond the DASS-21 scale used here. Moreover, unconsidered variables such as pre-existing medical conditions, psychoactive substance use, and family dynamics might significantly influence the results, suggesting the need for more comprehensive future research. Therefore, although the results indicate a directly proportional effect between sleep disturbances and mood disorders, we cannot infer causality between these variables.

PSQI and DASS-21 are validated instruments commonly used in sleep and mental health research. Nevertheless, they have certain limitations regarding their applicability to night-shift healthcare workers. While the PSQI has been validated and performs a robust assessment of sleep quality, it does not accurately represent the sleep architecture of subjects with altered circadian rhythms or daytime sleep characteristics that are seen in these professionals. Although the DASS-21 is a well-established measure for capturing mood symptoms, it does not necessarily account for some of the unique occupational stressors experienced by our participants. These limitations notwithstanding, our tools serve as valuable measures of objective sleep quality and subjective mental health in this population.

The study used a cross-sectional design, which restricts the ability to infer causal relationships between sleep deprivation and mood disorders. The cross-sectional design captures data at a single point in time, making it difficult to analyze how sleep deprivation can contribute to the development or intensification of mood disorders over time. Thus, although it is possible to observe associations between the variables, it cannot be said with certainty that sleep deprivation directly causes the mood disorders observed. Therefore, for a more robust analysis, longitudinal studies are recommended, as they allow participants to be followed over time. This would help clarify the temporal relationship between sleep deprivation and mood disorders, as well as allow a more detailed investigation into the direction of the relationship between these variables.

In conclusion, our findings reinforce the existence of a correlation between sleep quality and the levels of stress, depression, and anxiety among health professionals working nights, especially among nursing technicians who showed higher rates of these disorders compared to other professional categories. Based on the evidence found, we recommend the implementation of specific interventions to improve the quality of sleep and the psychological well-being of this population, which faces high levels of physical and emotional demand. Among the suggested interventions, we highlight the promotion of sleep hygiene programs to educate health professionals about the importance of maintaining a regular sleep routine and the adoption of practices that favor sleep quality, such as the creation of adequate and quiet resting environments within health units. In addition, CBT for insomnia could be made available as part of a psychological support program, helping these professionals develop strategies for dealing with the challenges of sleep and working night shifts.

From a health policy perspective, we suggest that hospital managers consider shift reorganization strategies that avoid long consecutive periods of night work and allow adequate recovery breaks between shifts. It is also recommended that health administrators evaluate the workload and number of shifts assigned to each professional, with the aim of minimizing overload and reducing the risks associated with sleep deprivation and mood disorders. Finally, it is essential that health policymakers consider including ongoing psychological support programs in the workplace, with an emphasis on assistance for professionals working night shifts, in order to prevent and treat the symptoms of stress, anxiety, and depression. Although this study provides valuable insights, it should be interpreted with caution due to the small sample size and the possibility of a response bias since the data were collected via an electronic questionnaire. Future research should consider a longitudinal design to investigate the causal relationship between sleep deprivation and mood disorders and explore other factors that influence mental health and sleep in healthcare professionals, such as pre-existing medical conditions, psychoactive substance use, and family dynamics. In this way, it will be possible to obtain more robust data and target increasingly effective interventions to promote the well-being of these essential professionals.
