# Supplementary material for: The relationship between sleep deprivation and the worsening of mood disorders in health professionals working night shifts
Source: Dement Neuropsychol. 2025 May 19;19:e20240186. doi: 10.1590/1980-5764-DN-2024-0186 (PMC12088667; doi:10.1590/1980-5764-DN-2024-0186)
Supplement: Supplementary file 1 [file 1980-5764-DN-19-e20240186-Suppl01.docx]

**SUPPLEMENTARY MATERIAL**

**Pittsburgh Sleep Quality Index**

The following questions relate to your sleeping habits over the last month only. Your answers should indicate the most accurate recollection of most days and nights in the last month. Please answer all questions.

Name:

Age:

Date:

1. During the last month, when did you usually go to bed at night?

Usual bedtime:

2. Over the last month, how long (in minutes) does it usually take you to fall asleep at night?

Number of minutes:

3. During the last month, when did you usually get up in the morning? Usual time to get up?

4. During the last month, how many hours of sleep did you get per night? (This may be different from the number of hours you stayed in bed) Hours of sleep per night:

5. During the last month, how often have you had trouble sleeping because you:

A) was unable to fall asleep within 30 minutes

1 = none in the last month

2 = less than once a week

3 = once or twice a week

4 = three or more times a week

B) woke up in the middle of the night or early in the morning

1 = none in the last month

2 = less than once a week

3 = once or twice a week

4 = three or more times a week

C) had to get up to go to the toilet

1 = none in the last month

2 = less than once a week

3 = once or twice a week

4 = three or more times a week

D) couldn't breathe comfortably

1 = none in the last month

2 = less than once a week

3 = once or twice a week

4 = three or more times a week

E) coughed or snored loudly

1 = none in the last month

2 = less than once a week

3 = once or twice a week

4 = three or more times a week

F) Felt very cold 1 = none in the last month

2 = less than once a week

3 = once or twice a week

4 = three or more times a week

G) felt very hot

1 = none in the last month

2 = less than once a week

3 = once or twice a week

4 = three or more times a week

H) had bad dreams

1 = none in the last month

2 = less than once a week

3 = once or twice a week

4 = three or more times a week

I) had pain

1 = none in the last month

2 = less than once a week

3 = once or twice a week

4 = three or more times a week

J) other reasons, please describe: ____________________________

1 = none in the last month

2 = less than once a week

3 = once or twice a week

4 = three or more times a week

6. Over the last month, how would you rate the quality of your sleep in general?

Very good

Good

Bad

Very bad

7. During the last month, how often have you taken medication (prescribed or on your own) to help you?

1 = none in the last month

2 = less than once a week

3 = once or twice a week

4 = three or more times a week

8. In the last month, how often have you found it difficult to stay awake while driving, eating or taking part in a social activity (party, meeting friends)?

1 = none in the last month

2 = less than once a week

3 = once or twice a week

4 = three or more times a week

9. Over the last month, how difficult has it been for you to keep up the enthusiasm to do things (your usual activities)? No difficulty at all

A minor problem

A reasonable problem

A big problem

10. Do you have a partner, spouse or roommate?

A) No

B) Partner or colleague, but in another room

C) Partner in the same room, but in a different bed

D) Partner in the same bed

If you have a partner or roommate, ask them how often in the last month you've presented:

E) Heavy snoring

1 = none in the last month

2 = less than once a week

3 = once or twice a week

4 = three or more times a week

F) Long pauses in breathing while asleep

1 = none in the last month

2 = less than once a week

3 = once or twice a week

4 = three or more times a week

G) twitching or pulling of legs while asleep

1 = none in the last month

2 = less than once a week

3 = once or twice a week

4 = three or more times a week

D) episodes of disorientation or confusion during sleep

1 = none in the last month

2 = less than once a week

3 = once or twice a week

4 = three or more times a week

E) Other changes (restlessness) while you sleep, please describe: ______________

1 = none in the last month

2 = less than once a week

3 = once or twice a week

4 = three or more times a week

#

# **DEPRESSION, ANXIETY AND STRESS SCALE (DASS)**

| Items | Answer options | | | |
| --- | --- | --- | --- | --- |
|  | It didn't apply at all | Applied to some degree or for some time | Applied to a considerable degree or for a good part of the time | Applied a lot, or most of the time |
| 1- I had trouble calming down | 0 | 1 | 2 | 3 |
| 2- I was aware that my mouth was dry | 0 | 1 | 2 | 3 |
| 3- I couldn't seem to get any positive feelings | 0 | 1 | 2 | 3 |
| 4- I had difficulty breathing (e.g. excessively rapid breathing, shortness of breath, in the absence of physical exertion) | 0 | 1 | 2 | 3 |
| 5- I found it difficult to take the initiative  things | 0 | 1 | 2 | 3 |
| 6- I tend to overreact to situations | 0 | 1 | 2 | 3 |
| 7- I felt tremors (e.g. in my hands) | 0 | 1 | 2 | 3 |
| 8- I felt that I was generally very nervous | 0 | 1 | 2 | 3 |
| 9- I worried about situations in which I might panic and look ridiculous | 0 | 1 | 2 | 3 |
| 10- I felt I had nothing to look forward to in the future | 0 | 1 | 2 | 3 |
| 11- I felt agitated | 0 | 1 | 2 | 3 |
| 12- I found it hard to relax | 0 | 1 | 2 | 3 |
| 13- I felt discouraged and depressed | 0 | 1 | 2 | 3 |
| 14- I was intolerant of things that prevented me from continuing what I was doing | 0 | 1 | 2 | 3 |
| 15- I felt like I was going to panic | 0 | 1 | 2 | 3 |
| 16- I couldn't get excited about anything | 0 | 1 | 2 | 3 |
| 17- I felt I didn't have much value as a person | 0 | 1 | 2 | 3 |
| 18- I felt sensitive | 0 | 1 | 2 | 3 |
| 19- I was aware of the functioning/beating of my heart in the absence of physical exertion (e.g. sensation of increased heart rate, cardiac dysrhythmia) | 0 | 1 | 2 | 3 |
| 20- I felt scared for no good reason | 0 | 1 | 2 | 3 |
| 21- I felt life was meaningless | 0 | 1 | 2 | 3 |
